# Stabilizing the cold plasma-stimulated medium by regulating medium’s composition

**DOI:** 10.1038/srep26016

**Published:** 2016-05-13

**Authors:** Dayun Yan, Niki Nourmohammadi, Ka Bian, Ferid Murad, Jonathan H. Sherman, Michael Keidar

**Affiliations:** 1 Department of Mechanical and Aerospace Engineering, The George Washington University, Science & Engineering Hall, 800 22^nd^ Street, NW, Room 3550, Washington, DC 20052, USA; 2 Department of Biological Sciences, The George Washington University, Lisner Hall, 2023 G Street, NW, Suite 340, Washington, DC 20052, USA; 3 Department of Biochemistry and Molecular Medicine, The George Washington University, Ross Hall 2300 Eye Street, NW, Washington, DC 20037, USA; 4 Neurological Surgery, The George Washington University, Foggy Bottom South Pavilion, 22^nd^ Street, NW, 7^th^ Floor, Washington, DC 20037, USA

## Abstract

Over past several years, the cold plasma-stimulated medium (PSM) has shown its remarkable anti-cancer capacity in par with the direct cold plasma irradiation on cancer cells or tumor tissues. Independent of the cold plasma device, PSM has noticeable advantage of being a flexible platform in cancer treatment. Currently, the largest disadvantage of PSM is its degradation during the storage over a wide temperature range. So far, to stabilize PSM, it must be remained frozen at −80 °C. In this study, we first reveal that the degradation of PSM is mainly due to the reaction between the reactive species and specific amino acids; mainly cysteine and methionine in medium. Based on this finding, both H_2_O_2_ in PSM and the anti-cancer capacity of PSM can be significantly stabilized during the storage at 8 °C and −25 °C for at least 3 days by using phosphate-buffered saline (PBS) and cysteine/methionine-free Dulbecco’s Modified Eagle Medium (DMEM). In addition, we demonstrate that adding a tyrosine derivative, 3-Nitro-L-tyrosine, into DMEM can mitigate the degradation of PSM at 8 °C during 3 days of storage. This study provides a solid foundation for the future anti-cancer application of PSM.

Over the past decade, cold atmospheric plasma has shown a selective anti-cancer capacity both *in vitro*^1^,^2^,^3^,^4^,^5^,^6^,^7^,^8^ and *in vivo*^9^,^10^,^11^,^12^,^13^. Various type of cold plasma devices were used to directly irradiate cancer cells cultured in the multi-well plates^4^,^14^, petri-dishes^1^,^15^, or tumor tissues^9^,^12^. Recently, plasma-stimulated medium (PSM) proved to exhibit a significant anti-cancer capacity as strong as the direct cold plasma treatment on glioblastoma cells^16^,^17^,^18^, lung carcinoma cells^19^, and bladder cancer cells^20^. Very recently, it is reported that microsecond-pulsed plasma-activated media is able to selectively inhibit the growth of lung cancer (H460) cells rather than normal lung cancer (L132) cells^21^. Another very recent study about the selective apoptosis in the PSM treated glioblastoma cells^22^, further confirming that PSM is a selective anti-cancer tool. The injection of PSM into mice also significantly inhibits the growth of tumor^23^. Thus, PSM may have wide application in cancer treatment including specific situations where cold atmospheric plasma cannot reach deep seated tumors or when the cold plasma device is not portable.

The cold plasma-originated reactive species are thought to be the main factor in cancer cell death and growth inhibition^24^. In fact, the feasibility of using PSM to kill cancer cells is a direct evidence to support this conclusion. When cold plasma interacts with the medium both reactive oxygen species (ROS) such as hydroxyl free radicals (OH)^25^ and hydrogen peroxide (H_2_O_2_)^19^,^26^ and reactive nitrogen species (RNS) such as nitric oxide (NO)^27^ and nitrite (NO_2_^−^)^17^,^28^ are dissolved in the aqueous solution. Among them, H_2_O_2_ has been found to mainly contribute to the death of cancer cells after the direct cold plasma irradiation on cancer cells^29^,^30^,^31^ or the indirect cold plasma irradiation on the culture medium^18^,^19^.

To date, the degradation of PSM during the storage is its largest disadvantage for future clinical application. For pharmaceutical reasons, PSM should be stably stored for relative long time. However, as it is known PSM gradually loses its anti-cancer capacity during the storage at the room temperature^17^,^19^ or down to the temperature a few degrees above the freezing point of water^19^. Accordingly, the H_2_O_2_ concentration in PSM also gradually decreases during the storage under these conditions^19^. Because the degradation of the anti-cancer capacity of PSM has been reported by different groups using different plasma devices and treatment doses^17^,^19^, the instability of PSM during the storage can be regarded as a basic feature of PSM.

So far, the sole strategy to inhibit the degradation of PSM during the storage is freeze at an adequately low temperature. Tetsuo Adachi *et al*. reported that a freeze at −80 °C was able to stabilize the anti-cancer capacity of cold plasma-stimulated Dulbecco’s Modified Eagle Medium (DMEM) for a week^19^. However, we found that freezing PSM at around −20 °C would significantly accelerate the degradation of PSM^17^. In fact, Tetsuo Adachi *et al*. also reported that the noticeable degradation of PSM still existed during the storage at −30 °C^19^. These observations indicate that the degradation of PSM during the storage may be due to a slow temperature-dependent reaction which can be inhibited in an adequately cold atmosphere. Nonetheless, according to the description of most manufacturers of cell culture media, the ideal storage temperature range for medium is over 2 °C to 8 °C, rather than the freezing condition. Thus, considering the clinical application prospective, PSM at least should be stable at such a temperature range. So far, no method has been reported about improving the stability of PSM over 2 °C to 8 °C.

In this study, by comparing the H_2_O_2_ concentration in the cold plasma-stimulated phosphate-buffered saline (PBS) and the cold plasma-stimulated DMEM during the storage at 8 °C, 22 °C and −25 °C, we found that the degradation of PSM was mainly due to the reaction between the plasma-originated reactive species and components in DMEM. A further detailed investigation showed that cysteine and methioine were the main components in DMEM contributing to this process. Based on these principles, we further proved that the plasma-stimulated cysteine/methionine-free DMEM was much more stable than the plasma-stimulated standard DMEM during the storage at 8 °C, 22 °C and −25 °C. In addition, adding a tyrosine derivative, 3-Nitro-L-tyrosine, could also improve the stability of cold plasma-stimulated DMEM at 8 °C but not at −25 °C.

## Results

**Figure 1:**
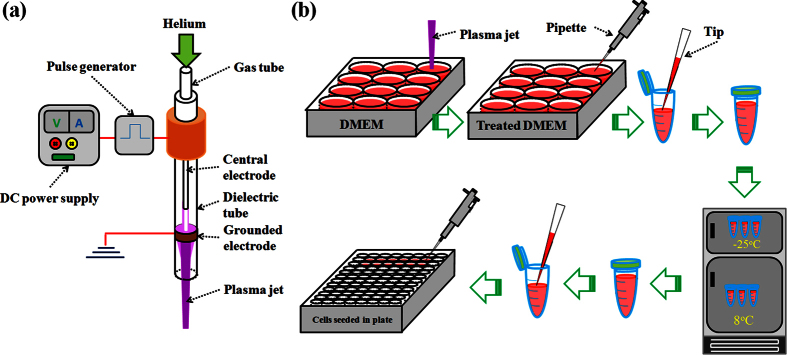
The cold plasma device (**a**) and the general research strategy using PSM to affect cancer cells seeded in multi-well plate (**b**).

The cold plasma device (Fig. 1a) was a typical helium cold atmospheric plasma jet generator which has been used in a series of studies^17^,^18^,^32^,^33^,^34^. Cold plasma jet was generated between the central electrode and the grounded ring electrode. The carrying gas was helium with a flow of 4.7 L/min and was controlled by a flow meter. The output voltage was 3.16 kV. The general research strategy is illustrated in Fig. 1b. 1 mL of PSM was made by the vertical irradiation on the medium in a well on a 12-well plate. Then, PSM was transferred into a 1.5 mL of centrifuge tube by pipette. The centrifuge tubes were stored in refrigerators with different internal temperatures (8 °C and −25 °C) for 3 days. Similarly, the centrifuge tubes were stored at 22 °C when we investigated the degradation of PSM at the room temperature (not shown in Fig. 1b). Ultimately, PSM was either transferred to culture the seeded cancer cells in a 96-well plate or transferred into a black-wall 96-well plate to perform the measurement for H_2_O_2_. The detailed protocols for the transfer are illustrated in Methods and Fig. S1a,b. Because PBS is not suitable for cell culture, the protocols used to transfer the plasma-stimulated PBS is slightly different from the protocols used to transfer the plasma-stimulated DMEM (Fig. S1).

**Figure 2:**
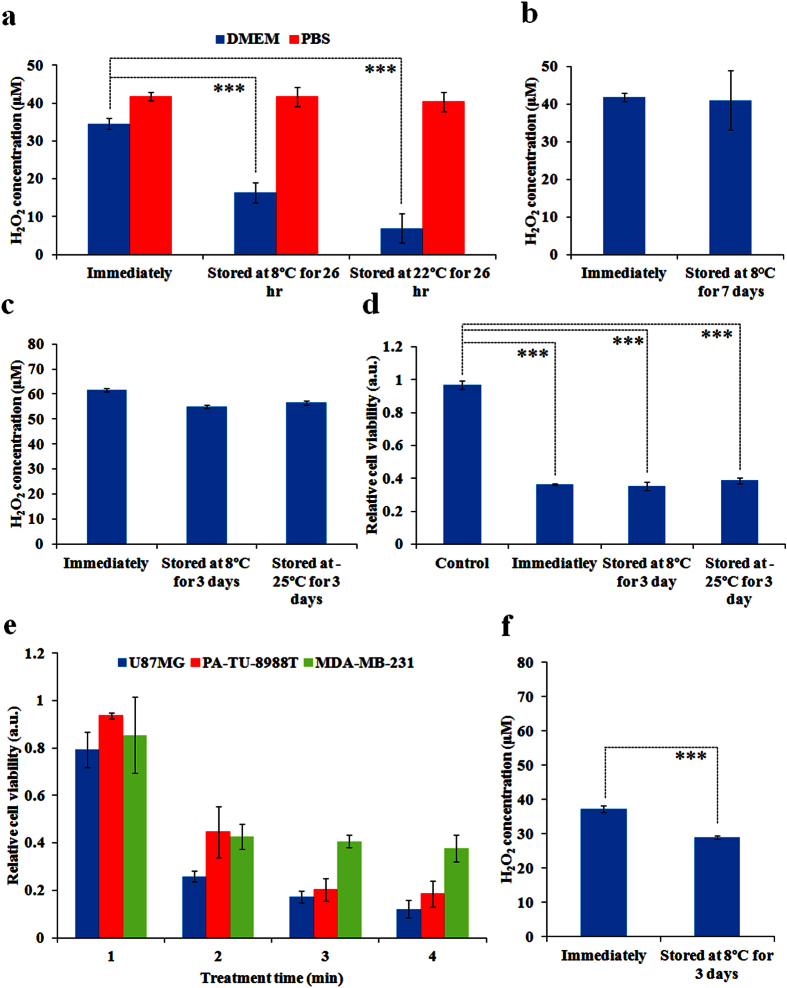
The plasma-stimulated PBS is a stable and effective anti-cancer tool. (**a**) The change of H_2_O_2_ concentration in the cold plasma-stimulated DMEM and PBS during the storage at 8 °C and 22 °C for 26 hours. (**b**) The change of H_2_O_2_ concentration in the cold plasma-stimulated PBS during the storage at 8 °C for 7 days. (**c**) The change of H_2_O_2_ concentration in the cold plasma-stimulated PBS during the storage at 8 °C and −25 °C for 3 days. (**d**) The change of anti-cancer capacity of the cold plasma-stimulated PBS during the storage at 8 °C and −25 °C for 3 days. (**e**) The effect of the cold plasma-stimulated PBS on the cell viability of U87MG, PA-TU-8988T, and MDA-MB-231 cells cultured in the cold plasma-stimulated PBS. (**f**) The change of H_2_O_2_ concentration in 37.3 μM H_2_O_2_-containing PBS during the storage at 8 °C for 3 days. The volume of solution for each well was 1 mL for all experiments. The treatment time for (**a**–**d**) was 1min and 2 min, respectively. Results are presented as the mean ± s.d. of three independently repeated experiments performed in triplicate (**a–c,f**) or in sextuplicate (**d,e**). For the cell viability, the data have been normalized to corresponding control group.Student’s t-test was performed and the significance is indicated as ***p < 0.005.

To understand the mechanism of PSM degradation, we first compared the H_2_O_2_ generation in the cold plasma-stimulated DMEM and PBS after 26 hours of storage and found that the H_2_O_2_ in plasma-stimulated PBS was quite stable during the storage while most H_2_O_2_ in the plasma-stimulated DMEM had been lost after 26 hours at 8 °C and 22 °C (Fig. 2a). The higher storage temperature for PSM, the stronger degradation occurs in PSM (Fig. 2a). Obviously, the instability of PSM is mainly due to the reaction between component of DMEM and reactive species generated by the cold plasma. Recently, we have demonstrated that the H_2_O_2_ in PSM tended to react with cysteine among all 20 amino acids after the plasma irradiation^18^. Thus, the plasma-stimulated PBS has higher H_2_O_2_ concentration than the plasma-stimulated DMEM (Fig. 2a). We further found that the H_2_O_2_ concentration in the plasma-stimulated PBS was not only stable over 7 days of storage at 8 °C (Fig. 2b) but also stable over 3 days at −25 °C (Fig. 2c). The stable anti-cancer capacity of the plasma-stimulated PBS over 3 days at 8 °C and −25 °C was further confirmed by using the corresponding treated PBS to affect the growth of breast cancer (MDA-MB-231) cells (Fig. 2d). In addition, we demonstrated that the plasma-stimulated PBS could also effectively inhibit the growth of glioblastoma (U87MG) cells and pancreatic cancer (PA-TU-8988T) cells with a dose-dependent manner (Fig. 2e), making it a stable anti-cancer PSM over a wide temperature range. Ultimately, we tested whether the H_2_O_2_-containing PBS would be as stable as the cold plasma-stimulated PBS, and found that the H_2_O_2_-containing PBS just experienced a slight degradation over the storage at 8 °C for 3 days (Fig. 2f), which is similar to the slight degradation observed in the plasma-stimulated PBS (Fig. 2c). The H_2_O_2_ in PBS will experience a natural degradation during the storage at 8 °C. Thus, the gradual H_2_O_2_ consumption in the cold plasma-stimulated DMEM is due to the combined effect of the natural degradation of H_2_O_2_ in aqueous solution and the reaction between H_2_O_2_ and the component in DMEM. The latter mainly controls the degradation at 8 °C.

**Figure 3:**
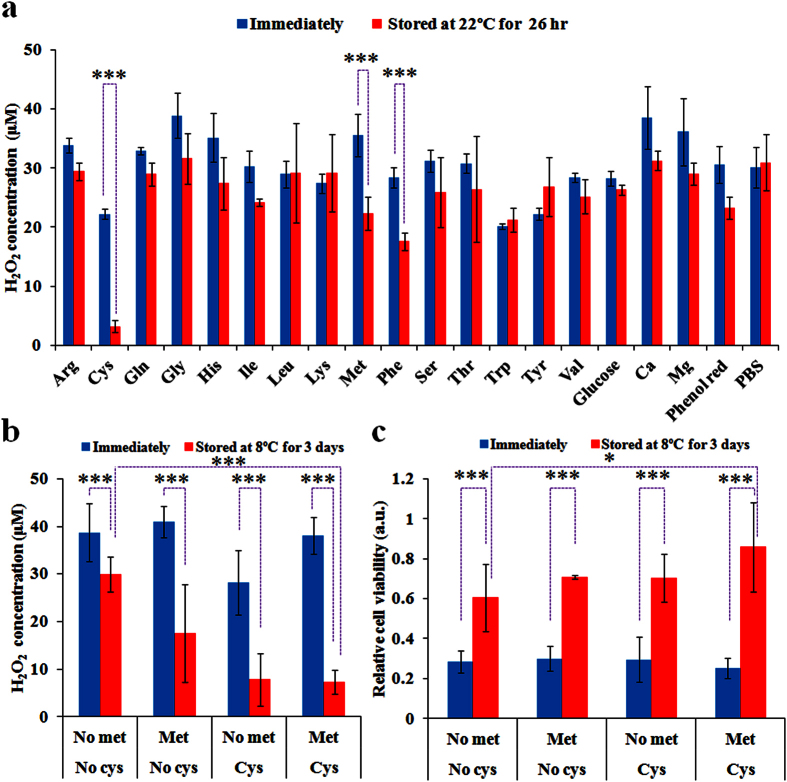
Cysteine and methionine mainly contribute to the degradation of the plasma-stimulated DMEM at 22 °C and 8 °C. (**a**) The change of H_2_O_2_ concentration in the cold plasma-stimulated PBS containing specific component in DMEM during the storage at 22°C for 26 hours. (**b**) The change of H_2_O_2_ concentration in the cold plasma-stimulated cysteine/methionine-free DMEM, cysteine-free DMEM, methionine-free DMEM, and standard DMEM during the storage at 8 °C for 3 days. (**c**) The change of anti-cancer capacity of the cold plasma-stimulated cysteine/methionine-free DMEM, cysteine-free DMEM, methionine-free DMEM, and standard DMEM during the storage at 8°C for 3 days. For all experiments, the volume of solution and the treatment time in each well was 1 mL and 1 min, respectively. Results are presented as the mean ± s.d. of three independently repeated experiments performed in triplicate (**a,b**) or in sextuplicate (**c**). For the cell viability, the data have been normalized to corresponding control group. Student’s t-test was performed and the significance is indicated as *p < 0.05, **p < 0.01, ***p < 0.005.

Furthermore, the effect of specific components in DMEM on the PSM degradation was comprehensively investigated. A comparison between DMEM and PBS reveals that 15 amino acids, glucose, calcium ion, magnesium ion, and phenol red are the main composition difference between DMEM and PBS (Fig. S2). Thus, 15 specific amino acids solutions, 1 glucose solution, 2 saline solutions (CaCl_2_, MgCl_2_), and 1 phenol red solution were respectively made by adding or dissolving specific component in PBS according to their concentrations in the standard DMEM (Fig. S3). After the plasma treatment and subsequent 26 hours of storage at 22 °C, the H_2_O_2_ concentrations in these solutions were measured and compared with corresponding H_2_O_2_ concentration in the solution just after the cold plasma treatment (Fig. 3a). The H_2_O_2_ in the plasma-stimulated PBS is quite stable over the storage. In contrast, most of the studied components cause different levels of H_2_O_2_ degradation in the plasma-stimulated PBS-based solution, except tryptophan, tyrosine, and lysine. Cysteine and methionine cause the most significant degradation of H_2_O_2_ during the storage at room temperature. Phenylalanine also cause the noticeably degradation of H_2_O_2_. Based on these data, it is reasonable to predict that a modified DMEM without cysteine, methionine, phenylalanine or other components will be a stable PSM. So far, however, the modified DMEM without phenylalanine is not available on the market. Thus, we just focused on studying the role of cysteine and methionine on the PSM degradation. We first compared the plasma-stimulated cysteine/methionine/glutamine-free DMEM with the plasma-stimulated arginine/lysine/glutamine-free DMEM and the plasma-stimulated standard DMEM and found that only the former was stable during the storage at 22 °C for 26 hours (Fig. S4). The absence of arginine and lysine did not inhibit the H_2_O_2_ degradation in PSM (Fig. S4). These results are consistent with the trends revealed in Fig. 3a.

To make cysteine/methionine-free DMEM, 4 mM glutamine was added in the purchased cysteine/methionine/glutamine-free DMEM. Based on these cysteine/methionine-free DMEM, we further prepared the modified DMEM just without cysteine and the modified DMEM just without methionine according to the description in Methods. Then, the specific effect of cysteine and methionine on the instability of PSM was investigated. After three days of storage at 8 °C, only the cold plasma-stimulated cysteine/methionine-free DMEM shows strong capacity inhibiting the H_2_O_2_ degradation (Fig. 3b). For cysteine and methionine, either of them will result in noticeable H_2_O_2_ degradation in PSM during the storage (Fig. 3b). Furthermore, the anti-cancer capacity of these PSM on breast cancer (MDA-MB-231) cells was investigated. In contrast to the experiments performed in the immediately treated medium, 3 days of storage at 8 °C causes noticeable degradation of the anti-cancer capacity of PSM even the modified DMEM does not contain cysteine and methionine (Fig. 3c). However, compared with other three cases, the cysteine/methionine-free DMEM still shows the strongest capacity to retain the effective species in PSM. The cysteine/methionine-free DMEM does not completely inhibit the H_2_O_2_ degradation, which may be due to two reasons. One is that many components other than cysteine and methionine in DMEM also contribute to the instability of PSM during the storage (Fig. 3a). The modified DMEM without all these reactive components may be as stable as PBS after the plasma irradiation. Another possibility is that some reactive species other than H_2_O_2_ may also contribute to the anti-cancer capacity of PSM.

**Figure 4:**
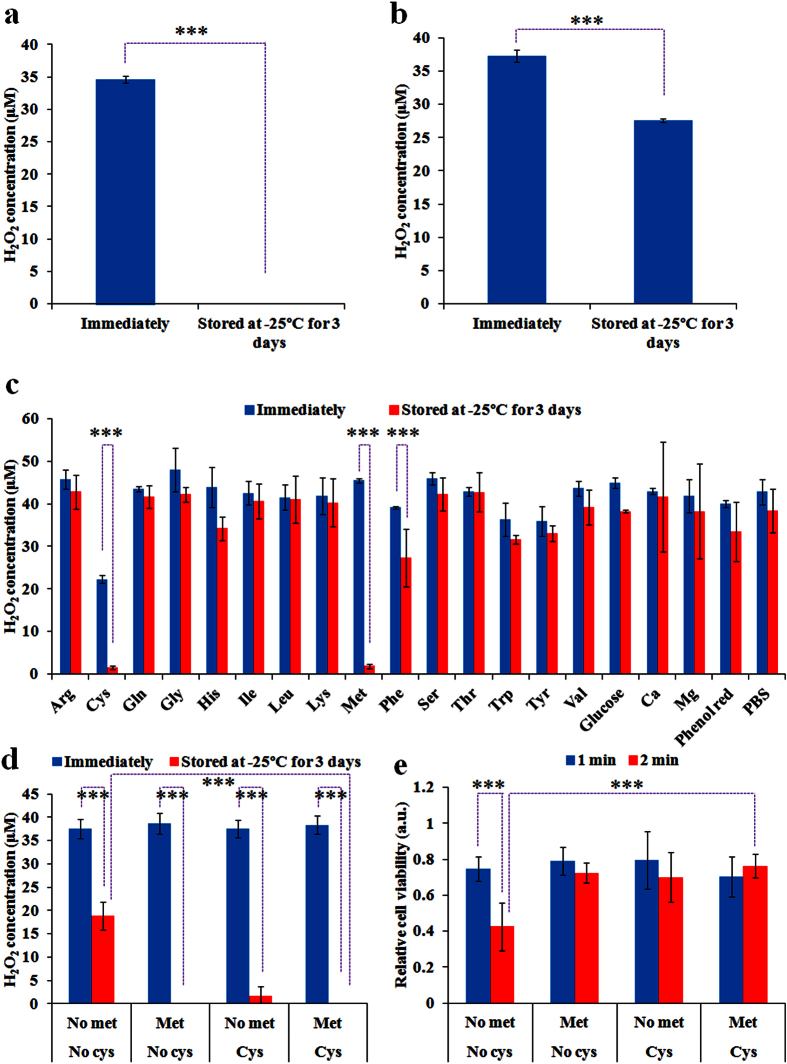
Cysteine and methionine mainly contribute to the degradation of the plasma-stimulated DMEM under the freezing condition. (**a**) The change of H_2_O_2_ concentration in 34.5 μM H_2_O_2_-containing DMEM during the storage at −25 °C for 3 days. (**b**) The change of H_2_O_2_ concentration in 37.3 μM H_2_O_2_-containing PBS during the storage at −25 °C for 3 days. (**c**) The change of H_2_O_2_ concentration in the cold plasma-stimulated PBS containing specific component in DMEM during the storage at −25 °C for 26 hours. (**d**) The change of H_2_O_2_ concentration in the cold plasma-stimulated cysteine/methionine-free DMEM, cysteine-free DMEM, methionine-free DMEM, and standard DMEM during the storage at −25 °C for 3 days. (**e**) The anti-cancer capacity of the cold plasma-stimulated cysteine/methionine-free DMEM, cysteine-free DMEM, methionine-free DMEM, and standard DMEM during the storage at −25 °C for 3 days. For all experiments, the volume of solution in each well was 1 mL. The treatment time was 1 min for (**c**,**d**). Results are presented as the mean ± s.d. of three independently repeated experiments performed in triplicate (**a–d**) or in sextuplicate (**e**). For the cell viability, the data have been normalized to corresponding control group. Student’s t-test was performed and the significance is indicated as ***p < 0.005.

Despite the optimized storage temperature for the culture medium should be between 2 °C to 8 °C, the cryopreservation of PSM may still be necessary at some special circumstances. For most of refrigerators served in pharmacies, the temperature in the freezing chamber is around −20 °C. To date, however, PSM has been found even more degradable when stored at temperature around −20 °C than that at 22 °C^17^ or at 4 °C^19^. General observation is that freezing aggravates the degradation of PSM. As we demonstrated above, both the H_2_O_2_ concentration (Fig. 2c) and the anti-cancer capacity (Fig. 2d) of the cold plasma-stimulated PBS are stably retained during the storage at −25 °C. Thus, the degradation of the plasma-stimulated DMEM should also be due to the reaction between component in DMEM and the plasma-originated reactive species such as H_2_O_2_. To verify this proposition, we first investigated the stability of H_2_O_2_-containing DMEM and H_2_O_2_-containing PBS under the freezing conditions for 3 days. It is found that, in contrast to the immediately prepared H_2_O_2_-containing DMEM and H_2_O_2_-containing PBS, H_2_O_2_ has been completely consumed (Fig. 4a) and has been well retained (Fig. 4b) in the frozen DMEM and the frozen PBS at −25 °C, respectively. Thus, the plasma-originated or other sources-originated H_2_O_2_ naturally tend to strongly react with specific component in DMEM under the freezing conditions. In addition, similar to the trend shown in Fig. 2f, H_2_O_2_ also shows a natural tend to be slightly consumed in PBS during the freezing storage. Thus, the degradation of H_2_O_2_ in PSM under the freezing condition is also due to the reaction between H_2_O_2_ and the components in media and a natural degradation in aqueous solution. The former is the main factor.

Similar to the research strategy described above, we further investigated which component in DMEM contribute to the degradation of PSM during the freezing storage. This was done by comparing the degradation of H_2_O_2_ in 15 amino acids solutions, 1 glucose solution, 2 saline solutions (CaCl_2_, MgCl_2_), 1 phenol red solution, and PBS. These solutions were made by previously mentioned methods according to the concentration shown in Fig. S3. As presented in Fig. 4c, cysteine and methionine are still the two most reactive components in DMEM consuming the plasma-originated H_2_O_2_ during the freezing storage. Different from the trend shown in Fig. 3a, all studied components tend to aggravate the degradation of H_2_O_2_ in the cold plasma-stimulated PBS at −25 °C, including tyrosine, tryptophan, and lysine, which in fact inhibit the degradation of H_2_O_2_ in the cold plasma-stimulated PBS at 8 °C (Fig. 3a). Under the freezing conditions, the H_2_O_2_ degradation in the methionine solution is stronger than that in the cysteine solution. Furthermore, we investigated the anti-degradation effect of the cysteine/methionine-free DMEM under the freezing conditions using the above mentioned methods. The appearance of either of cysteine and methionine in DMEM will result in the complete consumption of H_2_O_2_ after 3 days of storage at −25 °C (Fig. 4d). The cysteine-free DMEM is slightly more resistant to the H_2_O_2_ degradation than the methionine-free DMEM, which is consistent with the trend revealed in Fig. 4c that methionine is more reactive with H_2_O_2_ than cysteine during the freezing storage. For the cysteine/methionine-free DMEM, more than half of H_2_O_2_ has been consumed during the 3 days of storage at −25 °C, which is also consisted with trend shown in Fig. 4c that many other components in DMEM also contribute to the instability of PSM. Ultimately, we compared the anti-cancer capacity of these modified DMEM on MDA-MB-231 cells. When the plasma treatment time is just 1 min (Fig. 4e), the difference between four experimental groups is not obvious. Such phenomenon may be due to the low concentration of residual H_2_O_2_ or reactive species in frozen plasma-stimulated cysteine/methionine-free DMEM, which are too weak to cause noticeable anti-cancer effect (Fig. 4d). When the plasma treatment time extends to 2 min (Fig. 4e), the appearance of cysteine or methionine will significantly aggravate the degradation of the anti-cancer capacity of PSM. In contrast, the plasma-stimulated cysteine/methionine-free DMEM still causes a 60% of anti-cancer growth effect.

**Figure 5:**
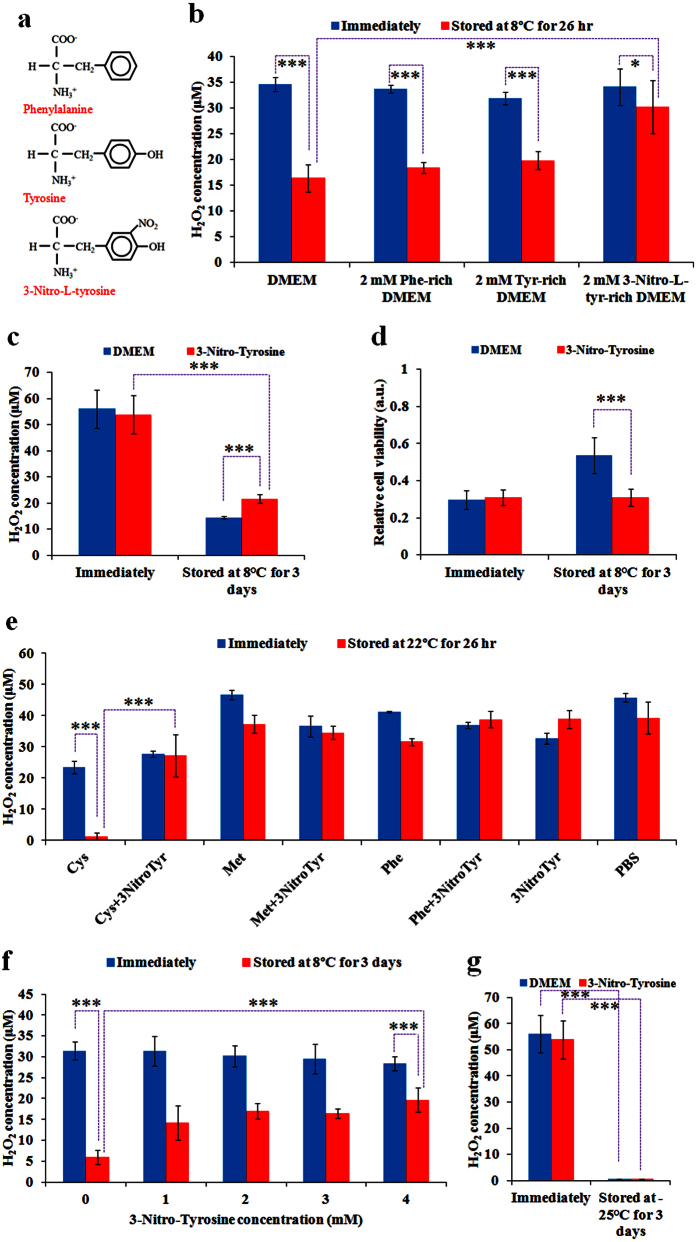
3-Nitro-L-tyrosine inhibits the degradation of cold plasma-stimulated DMEM. (**a**) Chemical formulas of phenylalanine, tyrosine, and 3-Nitro-L-tyrosine. (**b**) The change of H_2_O_2_ concentration in the cold plasma-stimulated standard DMEM, phenylalanine-containing DMEM, tyrosine-containing DMEM, and 3-Nitro-L-tyrosine-containing DMEM during the storage at 8 °C for 26 hours. (**c**) The change of H_2_O_2_ concentration in the cold plasma-stimulated standard DMEM and 3-Nitro-L-tyrosine-containing DMEM during the storage at 8 °C for 3 days. (**d**) The change of anti-cancer capacity of the cold plasma-stimulated standard DMEM and 3-Nitro-L-tyrosine-containing DMEM during the storage at 8 °C for 3 days. (**e**) The change of H_2_O_2_ concentration in the cold plasma-stimulated single amino acids-containing PBS and double amino acids-containing PBS during the storage at 22 °C for 26 hours. Corresponding concentration of cysteine, methionine, phenylalanine, and 3-Nitro-L-tyrosine in PBS were 0.2 mM, 0.2 mM, 0.4 mM and 2 mM, respectively. (**f**) The change of H_2_O_2_ concentration in the cold plasma-stimulated 3-Nitro-L-tyrosine-containing DMEM with different concentrations during the storage at 8 °C for 3 days. (**g**) The change of H_2_O_2_ concentration in the cold plasma-stimulated standard DMEM and 3-Nitro-L-tyrosine-containing DMEM during the storage at −25 °C for 3 days. For all experiments, the volume of solution in each well was 1 mL. The treatment time was 1 min for (**b,e,f**) and was 2 min for (**c,d,g**), respectively. Results are presented as the mean ± s.d. of three independently repeated experiments performed in triplicate (**b,c,e–g**) or in sextuplicate (**d**). For the cell viability, the data have been normalized to corresponding control group. Student’s t-test was performed and the significance is indicated as *p < 0.05, **p < 0.01, ***p < 0.005.

In addition to above mentioned methods, we presented a third anti-degradation method based on the results shown in Fig. 3a. It is clear that tyrosine is able to slightly increase the H_2_O_2_ concentration in the plasma-stimulated PBS during the storage at 8 °C. Among 15 amino acids in DMEM, only tyrosine and phenylalanine have aromatic ring structures (Fig. 5a). However, phenylalanine causes noticeable H_2_O_2_ degradation in the cold plasma-stimulated PBS during the storage at 8 °C (Fig. 3a). The only difference between tyrosine and phenylalanine is a hydroxyl on the aromatic ring (Fig. 5a), indicating that the modification on the aromatic ring of amino acids may endow the amino acids with a capacity inhibiting the degradation of H_2_O_2_ during the storage at 8 °C. We utilized a tyrosine derivative, 3-Nitro-L-tyrosine, which had a nitro group on the aromatic ring of tyrosine (Fig. 5a). 2 mM 3-Nitro-L-tyrosine-containing DMEM, 2 mM phenylalanine-containing DMEM, and 2 mM tyrosine-containing DMEM were prepared by dissolving purchased 3-Nitro-L-tyrosine, phenylalanine, and tyrosine powders in the standard DMEM, respectively. Subsequently, the stability of H_2_O_2_ in the plasma-stimulated DMEM, tyrosine-containing DMEM, phenylalanine-containing DMEM, and 3-Nitro-L-tyrosine-containing DMEM stored at 8 °C were compared. 3-Nitro-L-tyrosine significantly inhibits the degradation of H_2_O_2_ in PSM (Fig. 5b). On the contrary, tyrosine and phenylalanine do not improve the stability of PSM (Fig. 5b). Furthermore, it is found that noticeable H_2_O_2_ degradation still occur in the cold plasma-stimulated 3-Nitro-L-tyrosine-containing DMEM and the cold plasma-stimulated standard DMEM after the storage at 8 °C for 3 days (Fig. 5c). Despite 60% of plasma-originated H_2_O_2_ has been lost during the storage, it is more effective than the plasma-stimulated standard DMEM, in which 74% of plasma-originated H_2_O_2_ has been lost during the 3 days of storage. Nonetheless, after the storage, the cold plasma-stimulated 3-Nitro-L-tyrosine-containing DMEM still shows a noticeable anti-breast cancer cells capacity as strong as the immediately treated 3-Nitro-L-tyrosine-containing DMEM (Fig. 5d). In contrast, the anti-cancer capacity of the plasma-stimulated standard DMEM significantly decays during the storage (Fig. 5d).

To reveal the anti-degradation mechanism of 3-Nitro-L-tyrosine in the plasma-stimulated DMEM, we first investigated whether 3-Nitro-L-tyrosine was able to inhibit the H_2_O_2_ degradation in 0.2 mM cysteine-containing PBS, 0.2 mM methionine-containing PBS, and 0.4 mM phenylalanine-containing PBS. As shown in Fig. 5e, 2 mM 3-Nitro-L-tyrosine is able to completely inhibit the H_2_O_2_ degradation in all three cold plasma-stimulated amino acids-containing PBS solutions at 22 °C. Thus, the cold plasma-stimulated DMEM may be stabilized by 3-Nitro-L-tyrosine through inhibiting the reaction between some components in DMEM such as cysteine, methionine, or phenylalanine with the cold plasma-originated H_2_O_2_. Furthermore, we studied the concentration-effect of 3-Nitro-L-tyrosine on its anti-degradation effect in the plasma-stimulated DMEM. For the plasma-stimulated 3-Nitro-L-tyrosine-containing DMEM, the residual H_2_O_2_ after 3 days of storage increases as the concentration of 3-Nitro-L-tyrosine increases from 1 mM to 4 mM (Fig. 5f). After 3 days of storage at 8 °C, 4 mM 3-Nitro-L-tyrosine-containing DMEM loses 30% of the H_2_O_2_ originated from the plasma treatment. In contrast, the standard DMEM loses 81% of the plasma-originated H_2_O_2_ (Fig. 5f). Finally, we identified that the degradation of H_2_O_2_ in the 3-Nitro-L-tyrosine-containing DMEM during the freezing storage is as extensive as that occurs in the standard DMEM after the plasma treatment (Fig. 5g). In short, adding 3-Nitro-L-tyrosine in DMEM weakens the degradation of PSM stored at 8 °C, though it cannot weaken the degradation of PSM stored at −25 °C.

## Discussion

Understanding the interaction between the cold atmospheric plasma and molecules in an aqueous solution builds the foundation of the plasma medicine. Current experimentation is mainly limited to studying chemical reactions between the cold plasma-originated reactive species and components in an aqueous solutions such as cell culture medium^35^. Among diverse chemicals, amino acids are not only the building blocks of all proteins involving in nearly all biological processes but also the main component in most cell culture media utilized in the studies *in vitro*^4^,^6^,^16^. The interaction between the cold plasma and the amino acids in culture media has been extensively investigated in past two years. A general investigation regarding the chemical modification and change of 20 amino acids after the cold plasma treatment has been performed via high-resolution mass spectrometry^35^. It was found that hydroxylation, nitration, sulfonation, disulfide formation and sulfoxidation were the main chemical modification of 14 amino acids upon the cold plasma irradiation^35^. In addition, sulfur-containing amino acids such as cysteine and methionine and aromatic amino acids such as tryptophan, phenylalanine, and tyrosine were preferentially consumed during the plasma treatment^35^. It was also demonstrated that proteins in FBS consume the reactive species generated by cold plasma treatment significantly^17^. Recently, we further demonstrated that among 20 amino acids, cysteine was most reactive to plasma produced species including H_2_O_2_^18^. These results clearly revealed that the plasma-originated reactive species will be partially consumed immediately after their formation in PSM. Furthermore, in this study, we proved that many components of medium particularly cysteine and methionine will slowly consume the plasma-originated reactive species such as H_2_O_2_ during storage. Thus, in designing the ideal PSM, cysteine and methionine should be avoided. Such slow reaction between reactive species and components in medium should be temperature-dependent, because it can be completely inhibited through an adequately low temperature freeze at −80 °C^19^.

The discovery of the anti-degradation property of 3-Nitro-L-tyrosine provides a promising strategy to improve the stability of PSM because the original component of DMEM does not need to be removed. Despite the fact that the chemical mechanism of such anti-degradation capacity is unknown yet, adding modifications to the aromatic ring of tyrosine or phenylalanine may be the general strategy to obtain new derivatives capable to preserve H_2_O_2_ and perhaps other plasma produced reactive species. In addition, the anti-degradation effect of 3-Nitro-L-tyrosine first reveals that the interaction between the cold plasma and media cannot be simply described as the consumption of plasma-originated reactive species such as H_2_O_2_ or the modification of component such as cysteine and methionine in the medium. Indeed, most of components in the medium tend to consume the plasma-originated reactive species; however, specific amino acid derivatives are able to inhibit the reaction between plasma-originated reactive species and specific components in the medium such as cysteine and methionine. These results demonstrate that the chemical essence of interaction between cold plasma and aqueous solution is far from clear. We not only need to measure the immediate reaction in media due to the plasma irradiation but we also have to monitor the long term dynamic reactions in PSM after the plasma irradiation.

The essence of the anti-cancer reactive species in cold plasma or PSM is still disputable. A simple comparison between the concentration of H_2_O_2_ (Fig. 3b) and the anti-cancer effect (Fig. 3c) in the plasma-stimulated cysteine/methionine-free DMEM stored at 8 °C for 3 days reveals that though H_2_O_2_ just loses about 23% during the storage, corresponding anti-cancer capacity loses about 45% during the same storage. Perhaps other plasma-originated reactive species also contributes to the anti-cancer effect of PSM and decay faster than H_2_O_2_. Currently, H_2_O_2_ has just been regarded as the main plasma-originated reactive species contributing to the anti-cancer capacity of cold plasma^29^,^30^. However, evidence exist that both cellular-level^18^,^26^,^27^ and molecular-level^29^,^36^ response of cancer cells to the cold plasma treatment are different from that positive control using H_2_O_2_. The cold plasma-originated atomic oxygen has been proven to be a main toxic reactive species to epithelial kidney cells under a specific plasma treatment condition^37^. So far, based on the accumulated data in the literature, it is premature to reach final conclusion regarding the mechanism of action of cold atmospheric plasma. A further comprehensive investigation on the role of key plasma-originated reactive species on the anti-cancer effect of cold plasma is necessary and urgent.

## Conclusions

The degradation of PSM during storage is due to the reaction between the plasma-originated reactive species and components of the cell culture medium. We have found that cysteine and methionine are two most reactive components in DMEM contributing to the consumption of plasma-originated H_2_O_2_. The cold plasma-stimulated PBS and the cold plasma-stimulated cysteine/methionine-free DMEM can be stably stored at 8 °C and −25 °C for at least 3 days. In addition, adding 3-Nitro-L-tyrosine into DMEM could weaken the degradation of the cold plasma-stimulated DMEM at 8 °C for 3 days. These stabilizing strategies build a solid foundation for the application of PSM in future cancer treatment.

## Methods

### Medium and cell cultures

Standard Dulbecco’s modified Eagle’s medium (11965-118), the modified cysteine/methionine/glutamine-free DMEM (21013-024), the modified arginine/lysine/glutamine-free DMEM (A14431-01) and PBS (14040-133) were purchased from Gibco Life Technologies. In addition, all DMEM and PBS were mixed with 1% (v/v) antibiotic (penicillin and streptomycin) (Life Technologies) before any experiments. Human glioblastoma (U87MG) cells and pancreatic cancer (PA-TU-8988T) cells were provided by Dr. Murad’s Lab at the George Washington University. Human breast cancer (MDA-MB-231) cells were provided by Dr. Zhang’s Lab at the George Washington University. All cancer cell lines were seeded with a confluence of 3 × 10^4^ cells/mL and a volume of 100 μL in each well on 96-well plate and were cultured for 6 hours in a complete media composed of Dulbecco’s modified Eagle’s medium (Life Technologies) supplemented with 10% (v/v) fetal bovine serum (ThermoFisher Scientific) and 1% (v/v) antibiotic (penicillin and streptomycin) solution (Life Technologies) under the standard cell culture conditions (a humidified, 37 °C, 5% CO_2_ environment). In each experiment, 6 wells in a single column on 96-well plate would be seeded with cancer cells.

### Making plasma-stimulated PBS and DMEM

In this study, the protocols to make PSM are the same among different experiments. 1 mL of PBS or DMEM or other modified DMEM such as cysteine/methionine-free DMEM in a well on 12-well plate (Falcon) was treated by cold plasma for 1 min or 2 min. The gap between the end of dielectric plasma tube and the bottom of 12-well plate was 3 cm.

### Making H_2_O_2_-containing PBS and DMEM

The H_2_O_2_-containing PBS and H_2_O_2_-containing DMEM were prepared by adding purchased 30 wt% H_2_O_2_ solution (Sigma-Aldrich) into PBS and DMEM, respectively. The H_2_O_2_ concentration in H_2_O_2_-containing PBS and H_2_O_2_-containing DMEM were close to the H_2_O_2_ concentration in the 1 min of cold plasma-treated 1 mL of PBS or DMEM in 12-well plate, respectively.

### Making the specific component-containing PBS and DMEM

The amino acids-containing PBS, the calcium-containing PBS, the magnesium-containing PBS, the glucose-containing PBS, the phenol red-containing PBS, and amino acids-containing DMEM were prepared by adding and dissolving purchased specific amino acids, calcium chloride solution, magnesium chloride solution, D-glucose and phenol red solution or amino acids derivative such as 3-Nitro-L-tyrosine into PBS and DMEM, respectively. Except PBS and DMEM, all above chemicals were purchased from Sigma-Aldrich.

### Affecting the growth of cancer cells seeded in 96-well plate by the cold plasma-stimulated DMEM or PBS

First, the initial culture medium which has been cultured cells for 6 hours has been removed before this step. Then, for the plasma-stimulated DMEM (Fig. S1a), 100 μL of treated DMEM was transferred from a well on 12-well plate or other tubes stored in refrigerator into a well on 96-well plate, in which 3 × 10^3^ cancer cells were seeded and has been cultured for 6 hours. In each experiment, 6 wells in a single column on 96-well plate have been seeded with cancer cells. In the control group, the DMEM used to culture cancer cells was the untreated DMEM. For the plasma-stimulated PBS (Fig. S1b), considering that cancer cells cannot grow in PBS over a long time range, 100 μL of untreated DMEM was first transferred into the well seeded with 3 × 10^3^ cancer cells on 96-well plate. Then, 100 μL of treated PBS was transferred into the well which contained 100 μL of untreated DMEM and 3 × 10^3^ cancer cells. Thus, after this step, the total volume of the mixed medium in each well seeded with cancer cells were 200 μL. For the control group, the PBS transferred into the well would not be treated. Ultimately, for above two cases, the cancer cells on 96-well plate were cultured under a standard cell culture condition (a humidified, 37 °C, 5% CO_2_ environment) for 3 days.

### Measuring and processing the cell viability

After the experiments, the cancer cells in 96-well plates were cultured in 96-well plate under the standard cell culture conditions (a humidified, 37 °C, 5% CO_2_ environment) for 3 days. Then, according to the standard two-steps MTT measurement protocols, the viability of cancer cells was qualified by MTT test and was read by an H1 microplate reader (Hybrid Technology) at the absorbance of 570 nm. In each independent experiment, 6 wells in a single column on 96-well plate would be seeded with cancer cells and would be taken cell viability test. The average value of measured cell viability from 6 wells was regarded as the cell viability measured from one independent experiment. To facilitate the understanding on data, all data about cell viability shown in figures has been normalized to the control group by dividing the measured cell viability of experimental group by the measured cell viability of control group. The final data shown in all figures are the mean ± s.d. of the normalized cell viability from three independently repeated experiments.

### Measuring H_2_O_2_ concentration

50 μL of medium or PBS which needs to be measured was transferred to a well on a black-wall 96-well clear bottom plate (Falcon) in triplicate. Next, according to the standard protocols provided by Sigma-Aldrich, the H_2_O_2_ concentration in the cold plasma-stimulated medium was measured. The fluorescence at 540/590 nm was read using an H1 microplate reader (Hybrid Technology).

## Additional Information

**How to cite this article**: Yan, D. *et al*. Stabilizing the cold plasma-stimulated medium by regulating medium's composition. *Sci. Rep.* **6**, 26016; doi: 10.1038/srep26016 (2016).

## Supplementary Material

Supplementary Information
