# Supplementary material for: Stabilizing the cold plasma-stimulated medium by regulating medium’s composition
Source: Sci Rep. 2016 May 13;6:26016. doi: 10.1038/srep26016 (PMC4865954; doi:10.1038/srep26016)
Supplement: Supplementary Information [file srep26016-s1.doc]

**Stabilizing the cold plasma-stimulated medium by regulating medium’s composition**

Dayun Yan^1*^, Niki Nourmohammadi^2^, Ka Bian^3^, Ferid Murad^3^, Jonathan H.Sherman^4^, and Michael Keidar^1*^

^1^Department of Mechanical and Aerospace Engineering, The George Washington University, Science & Engineering Hall, 800 22^nd^ Street, NW, Room 3550, Washington, DC 20052, USA

^2^Department of Biological Sciences, The George Washington University, Lisner Hall, 2023 G Street, NW, Suite 340, Washington, DC 20052, USA

^3^Department of Biochemistry and Molecular Medicine, The George Washington University, Ross Hall 2300 Eye Street, NW, Washington, DC 20037, USA

^4^Neurological Surgery, The George Washington University, Foggy Bottom South Pavilion, 22^nd^ Street, NW, 7^th^ Floor, Washington, DC 20037, USA

*Corresponding authors: Dayun Yan [ydy2012@gwmail.gwu.edu](mailto:ydy2012@gwmail.gwu.edu)

Michael Keidar [keidar@gwu.edu](mailto:keidar@gwu.edu)


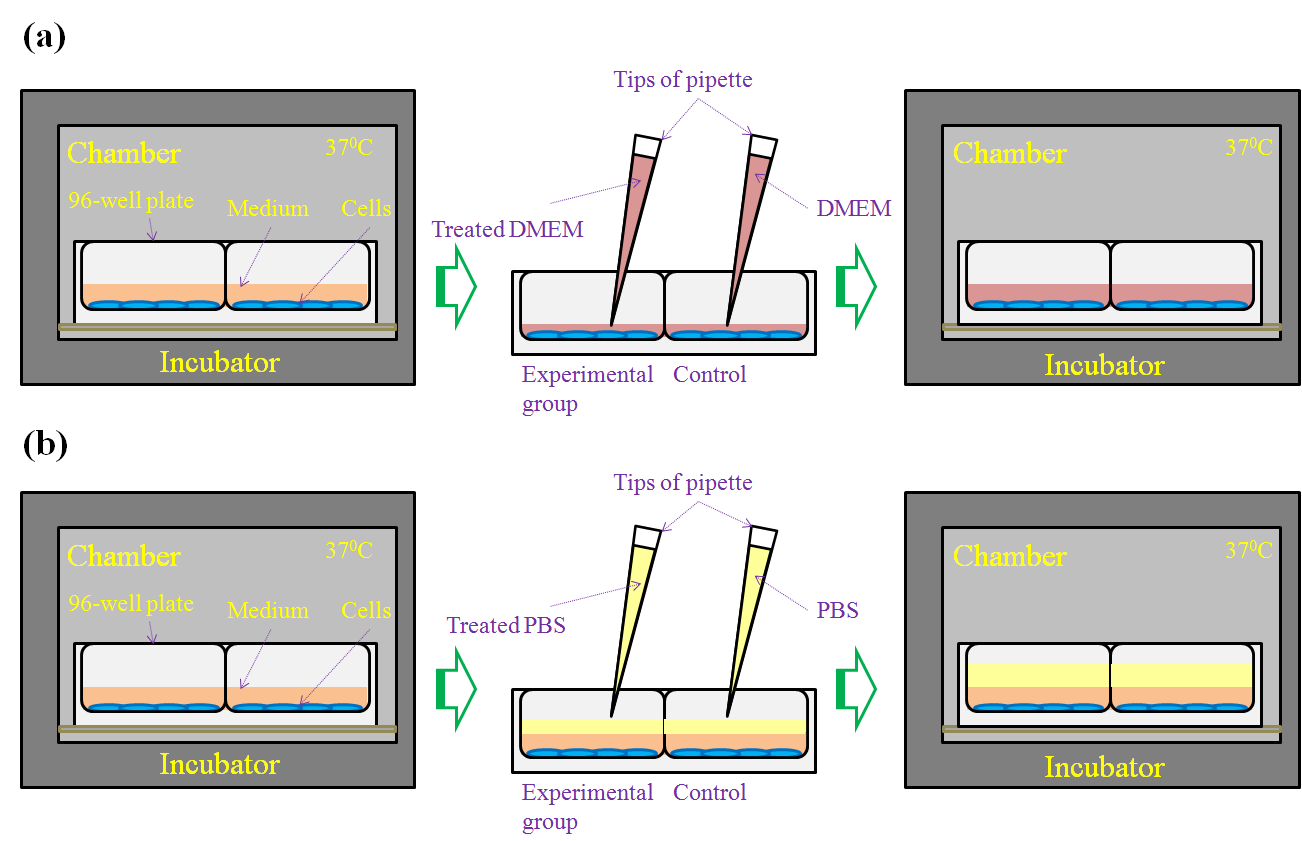


Figure S1. Schematic illustrations for affecting the growth of cancer cells seeded in a 96-well plate using the plasma-stimulated DMEM (a) or PBS (b). The detailed description is illustrated in **Methods**.


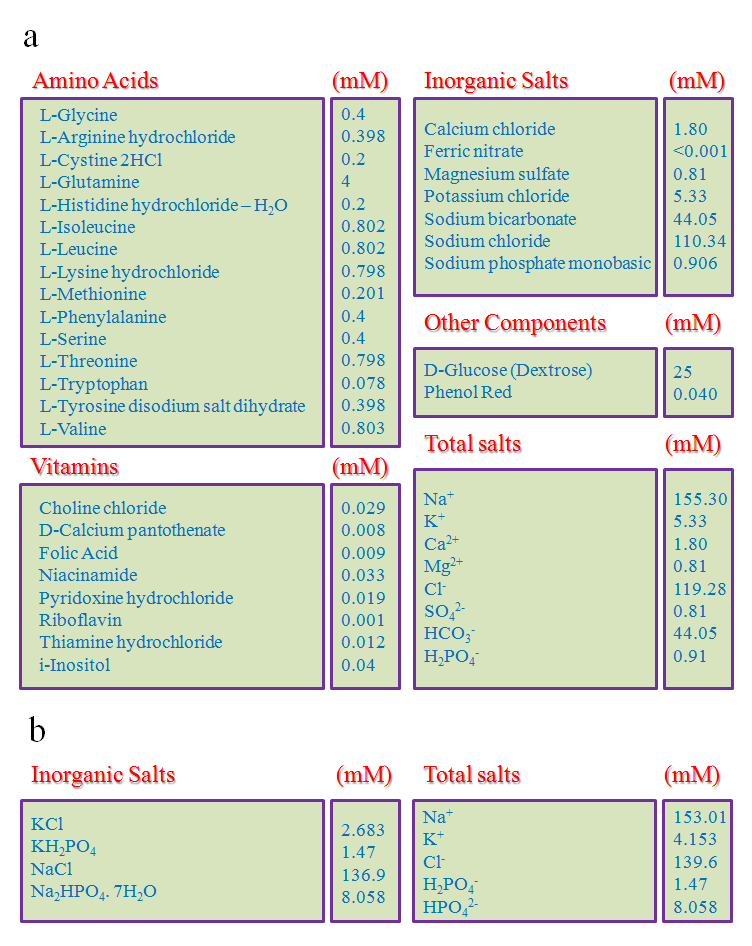


Figure S2. The composition of DMEM (11965-118, Gibco Life Technologies) (a) and PBS (14040-133, Gibco Life Technologies) (b).


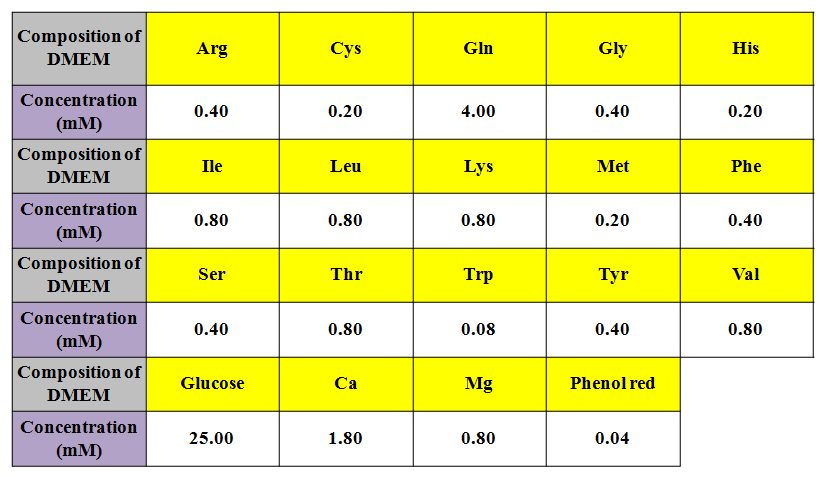


Figure S3. The main concentration of chemicals in DMEM (11965-118, Gibco Life Technologies)


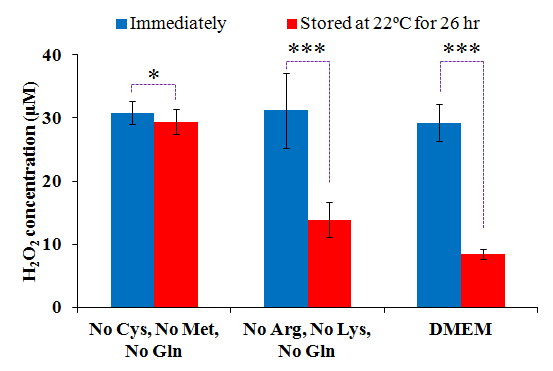


Figure S4. The change of H_2_O_2_ concentration in the plasma-stimulated cysteine/methionine/glutamine-free DMEM, arginine/lysine/glutamine-free DMEM, and standard DMEM after the storage at 22°C for 26 hours. For all experiments, the volume of solution and the treatment time in each well was 1 mL and 1 min, respectively. Results are presented as the mean ± s.d. of three independently repeated experiments performed in triplicate. Student’s t-test was performed and the significance is indicated as * p < 0.05, ** p< 0.01, *** p<0.005.
